# Employing Nutrition to Delay Aging: A Plant-Based Telomere-Friendly Dietary Revolution

**DOI:** 10.3390/nu17122004

**Published:** 2025-06-14

**Authors:** Joanna Polom, Virginia Boccardi

**Affiliations:** 1 Department of Medicine, The Academy of Applied Medical and Social Sciences, Lotnicza 2, 82-300 Elblag, Poland; 2 Department of Medical Laboratory Diagnostics-Fahrenheit Biobank BBMRI.pl, Medical University of Gdańsk, Marii Skłodowskiej-Curie 3a, 80-210 Gdańsk, Poland; 3 Division of Gerontology and Geriatrics, Department of Medicine and Surgery, University of Perugia, 06123 Perugia, Italy

**Keywords:** aging, diet, nutrition, plant, telomere

## Abstract

Telomere attrition is a hallmark of cellular aging, influenced by oxidative stress, chronic inflammation, and metabolic dysregulation. Emerging evidence suggests that dietary patterns rich in plant-based, minimally processed foods may influence telomere dynamics, potentially extending healthspan. This narrative review synthesizes current literature on the molecular mechanisms by which specific nutrients—such as antioxidants, polyphenols, omega-3 fatty acids, and methyl donors—affect telomere length and telomerase activity. Conversely, high consumption of ultra-processed foods (UPFs) has been associated with accelerated telomere shortening and dysfunction, likely due to increased oxidative stress, inflammation, and nutrient deficiencies. We propose a tiered dietary intervention model including preventive, therapeutic, and regenerative phases, tailored to individual aging trajectories and physiological statuses. This model emphasizes the consumption of whole plant foods, functional bioactives, and the reduction of UPFs to preserve telomere integrity. Implementing such dietary strategies may offer a viable approach to mitigate age-related cellular decline and promote healthy aging.

## 1. Introduction

The aspiration of extended human longevity and enhanced healthspan has emerged as a central focus in biomedical and gerontological research, reflecting a paradigm shift from merely prolonging life to optimizing the quality of life during aging. Longevity, traditionally defined as the total duration of life, is now increasingly considered alongside healthspan, the period of life spent in good health, free from the chronic diseases and disabilities of aging [1]. A critical determinant of both longevity and healthspan lies at the cellular level, specifically within the dynamics of cellular longevity. Cellular longevity is related to the capacity of cells to maintain functional integrity and replicate without undergoing senescence or apoptosis. A pivotal component of cellular longevity is the preservation of genomic stability, in which telomeres—nucleotide repeats that cap chromosomal ends—play a vital role. Telomeres prevent genomic degradation during cell division, yet progressively shorten with each replication cycle, ultimately leading to replicative senescence once a critical length is reached [2]. This telomeric attrition is exacerbated by oxidative stress, inflammation, and metabolic dysregulation—processes intrinsically linked to diet and lifestyle [3]. Although many studies have explored associations between dietary patterns and telomere length, few have addressed population-specific responses or the interaction of diet with inflammatory phenotypes in older adults. Again, there is limited integration between dietary modulation of telomere biology and clinically validated aging markers [4,5]. Emerging evidence highlights the potential of specific nutritional strategies to modulate telomere dynamics, thereby influencing cellular aging. Plant-based nutrients have shown promise in preserving telomere attrition along with aging [6,7]. As such, the concept of a “telomere-friendly diet” can be adopted as a translational approach to promote cellular resilience and delay age-related decline [8]. In this context, this review proposes a comprehensive dietary framework grounded in current molecular and nutritional science aimed at optimizing telomere integrity. It synthesizes evidence on the molecular mechanisms by which diet influences telomere biology and outlines strategic nutritional interventions that may extend cellular longevity, thereby contributing to overall healthspan and lifespan extension.

## 2. Methodology

This review adopts a narrative, integrative review design aimed at synthesizing current knowledge regarding the role of nutrition—specifically plant-based and minimally processed diets—in modulating telomere dynamics and promoting cellular longevity. A comprehensive literature search was conducted across major biomedical databases including PubMed, Scopus, and Web of Science. Keywords included: “telomere length”, “telomerase activity”, “plant-based diet”, “processed foods”, “oxidative stress”, “inflammation”, “cellular senescence”, “aging”, and “nutritional interventions”. The inclusion criteria were as follows: (1) peer-reviewed articles; (2) studies published primarily between 2013 and 2025 to reflect the most recent advancements, while including earlier studies foundational to the field; (3) articles in English; and (4) studies focusing on human subjects or relevant in vitro/in vivo mechanistic insights related to telomere biology and dietary interventions. The review begins with an analysis of the molecular mechanisms of aging and the pivotal role of telomeres, then examines how specific nutrients and dietary patterns, particularly plant-based diets, influence telomere dynamics. The discussion then shifts to the detrimental effects of ultra-processed foods on cellular aging, culminating in the proposal of a “telomere-friendly” diet as an innovative strategy to promote healthy aging by preserving telomere length—the molecular hallmark often referred to as the body’s “biological clock”.

## 3. Telomere Dynamics and the Molecular Architecture of Aging

Aging represents a dynamic and complex biological phenomenon influenced by a combination of genetic predispositions, epigenetic modifications, and environmental exposures [1]. These elements collectively coordinate a range of molecular and cellular alterations that support the progressive deterioration of physiological systems and heighten vulnerability to chronic diseases typical of later life. Among the defining features of the aging process, the progressive shortening of telomeres—repetitive nucleotide sequences (TTAGGG in humans) at chromosome ends—has emerged as a critical factor in the regulation of cellular lifespan, genomic stability, and systemic aging [2]. Telomeres, along with the shelterin protein complex that shields them, serve as essential guardians of chromosomal integrity by preventing deleterious end-to-end fusions, aberrant recombination events, and loss of genetic information during cell division. Yet, due to the end-replication limitation of DNA polymerase, telomeres inexorably erode with successive rounds of mitosis. This replication-linked attrition is exacerbated by oxidative damage and chronic inflammation—two interrelated processes that disproportionately affect telomeric DNA owing to its high guanine content and reduced capacity for effective repair [9,10].

Reactive oxygen species (ROS), generated endogenously via mitochondrial respiration and exogenously through environmental insults, contribute to telomere damage and erosion [11]. Concomitantly, chronic low-grade inflammation—characterized by elevated levels of cytokines such as interleukin-6 (IL-6) and tumor necrosis factor-alpha (TNF-α)—amplifies cell turnover and perpetuates a pro-senescent cellular milieu [12]. The convergence of these processes, collectively termed “inflammaging”, is increasingly recognized as a fundamental mechanism driving accelerated telomere attrition and the pathogenesis of numerous age-associated disorders, including neurodegeneration, atherosclerosis, and metabolic syndromes [12,13].

In select cellular compartments, including germ cells, certain stem cell populations, and activated immune cells, the enzyme telomerase—a reverse transcriptase complex incorporating an RNA template—actively restores telomeric sequences, thereby maintaining chromosomal stability [14]. However, in most somatic tissues, telomerase expression is tightly repressed, rendering these cells susceptible to telomere-induced replicative senescence. Critically shortened telomeres elicit a robust DNA damage response (DDR), leading to permanent cell cycle arrest via activation of the p53–p21 and p16INK4a-Rb tumor suppressor pathways or, in some contexts, apoptotic cell death [15]. These senescent cells, although initially protective against oncogenic transformation, accumulate with age and contribute to tissue dysfunction through the secretion of pro-inflammatory and matrix-degrading factors—a phenotype known as the senescence-associated secretory phenotype (SASP). The net effect is a progressive decline in tissue regenerative potential and an increased predisposition to age-related pathology [16]. Recognizing the central role of telomere biology in aging and disease, emerging therapeutic strategies aim to preserve telomere length and enhance telomerase activity. Lifestyle and dietary interventions have shown promise in this regard. Diets enriched with polyphenols, antioxidants, omega-3 fatty acids, and anti-inflammatory nutrients are increasingly associated with longer telomeres and improved telomere maintenance [17]. These findings underscore the potential of nutritional strategies not only to delay the onset of aging-related conditions but also to promote healthy longevity.

## 4. Nutritional Modulation of Telomere Dynamics: Molecular Pathways and Mechanisms

Emerging preclinical and observational evidence highlights the potential role of nutrition in modulating telomere length and telomerase activity, thereby influencing cellular aging and healthspan. While these findings are promising, nutritional interventions remain investigational in the context of telomere-targeted strategies and require further clinical validation [17]. Nutrients impact telomere biology via several interrelated molecular mechanisms, notably by mitigating oxidative stress, modulating chronic inflammation, and influencing epigenetic pathways [18]. These mechanisms converge to preserve chromosomal stability, delay replicative senescence, and enhance the regenerative capacity of tissues [19]. Reactive oxygen species (ROS), primarily generated by mitochondrial respiration and inflammatory responses, accelerate telomere shortening by inducing DNA strand breaks [20]. Antioxidant-rich diets—such as those abundant in vitamins C and E, polyphenols, and carotenoids—scavenge ROS and upregulate endogenous antioxidant enzymes like superoxide dismutase (SOD), catalase, and glutathione peroxidase (GPx). These activities reduce the oxidative burden on telomeric DNA [20,21]. Chronic low-grade inflammation—or “inflammaging”—is a key contributor to telomere attrition [22]. Nutrients with anti-inflammatory properties, such as omega-3 fatty acids (EPA and DHA), curcumin, and flavonoids, suppress the nuclear factor-kappa B (NF-κB) signaling pathway and inhibit pro-inflammatory cytokines like IL-6, TNF-α, and C-reactive protein (CRP). By reducing systemic inflammation, these compounds indirectly preserve telomere length and reduce the SASP [21,22]. Telomerase, particularly its catalytic subunit hTERT, is regulated through epigenetic modifications including DNA methylation, histone acetylation, and microRNAs [23,24]. Certain bioactive dietary components—such as resveratrol, sulforaphane, and EGCG (epigallocatechin gallate)—modulate these epigenetic marks, resulting in the upregulation of hTERT expression in selected cell types [25]. Furthermore, folate and B vitamins serve as methyl donors in one-carbon metabolism, influencing DNA methylation patterns at telomerase and other aging-related genes [26]. Nutrient-driven of metabolic sensors such as AMP-activated protein kinase (AMPK), sirtuins (particularly SIRT1), and the mTOR pathway also impacts telomere integrity [27,28]. Caloric restriction and compounds that mimic its effects—like nicotinamide riboside and polyphenols—activate AMPK and SIRT1, enhancing mitochondrial biogenesis and reducing metabolic stress [29]. These actions lower ROS production and promote stability through indirect metabolic regulation [30,31].

A growing body of evidence suggests that specific nutrients and bioactive compounds can profoundly influence telomere maintenance (Table 1). Vitamin C, a potent antioxidant, has been shown in both preclinical studies and observational human data to scavenge ROS and enhance the activity of endogenous antioxidant enzymes such as SOD and GPx, thereby reducing oxidative damage to telomeric DNA [32]. Vitamin E also benefits from a robust evidence base, including large population-based observational studies, demonstrating its role in stabilizing cellular membranes and preventing lipid peroxidation, a critical factor in telomere protection [33]. Polyphenols, notably resveratrol, have been primarily studied in cell and animal models, with some emerging human data suggesting activation of SIRT1 and modulation of DNA methylation [34], potentially enhancing telomerase expression and telomere stabilization [35]. Omega-3 fatty acids are supported by randomized controlled trials demonstrating their capacity to inhibit NF-κB signaling and suppress inflammatory cytokines such as IL-6 and TNF-α, thereby mitigating inflammation-driven telomere attrition [36]. Curcumin, mainly investigated in preclinical and small-scale clinical studies, inhibits NF-κB activation and induces the expression of protective genes, which may shield telomeres from inflammatory erosion [37]. Flavonoids, supported by a growing body of preclinical and epidemiological research, modulate cytokine production and attenuate the SASP, contributing to telomere preservation [38]. Folate and B vitamins, essential for one-carbon metabolism and DNA methylation, are implicated in epidemiological studies as supporting the transcription of hTERT, influencing telomere elongation and cellular homeostasis [39]. EGCG, the major catechin in green tea, has been studied in preclinical models where it alters histone acetylation and DNA methylation patterns, thereby facilitating hTERT upregulation and promoting epigenetic stability [40,41]. Sulforaphane, through activation of the Nrf2 pathway and inhibition of histone deacetylases, shows compelling preclinical evidence for enhancing cellular detoxification and reducing oxidative stress, contributing to telomere maintenance [42]. In contrast, nicotinamide riboside, though promising in mechanistic and animal studies, currently lacks substantial human clinical evidence directly linking it to telomere preservation. Its proposed benefits derive from NAD+-mediated activation of AMPK and SIRT1, supporting mitochondrial health and reducing ROS production [43], and indirectly stabilizing telomeres. Collectively, these findings support a nutritionally driven strategy for the maintenance of telomere integrity, with potential implications for delaying cellular senescence and promoting healthy aging, underscoring the importance of dietary and lifestyle interventions in longevity research.

**Figure 1:**
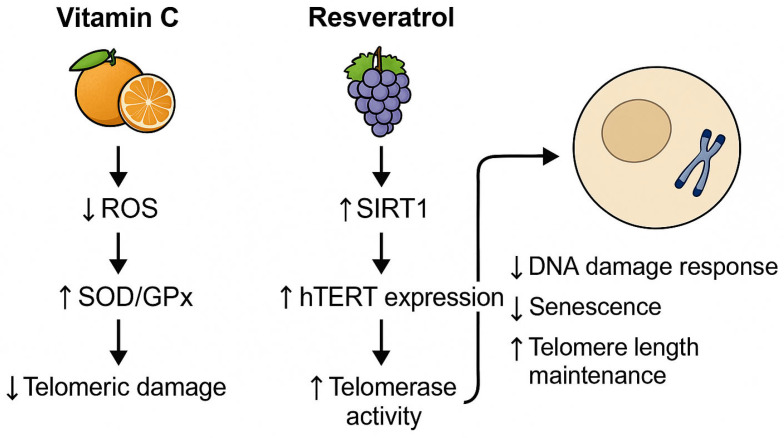
Nutrient-modulated mechanisms supporting telomere integrity. ROS = reactive oxygen species; SOD = superoxide dismutase; GPx = glutathione peroxidase; SIRT1 = sirtuin 1; hTERT = human telomerase reverse transcriptase.

To illustrate the mechanistic link between diet and telomere integrity, we elaborate on two representative nutrients with well-documented molecular effects. Vitamin C, a potent antioxidant, reduces telomeric DNA damage by neutralizing ROS and enhancing endogenous enzymatic defense systems such as SOD and GPx. This reduces oxidative stress at guanine-rich telomere sequences, thereby decelerating attrition. Conversely, resveratrol, a polyphenol found in grapes and berries, activates SIRT1, a NAD+-dependent deacetylase, and modulates epigenetic marks on the promoter of hTERT, the catalytic subunit of telomerase. This enhances telomerase expression and elongation, particularly in immune and endothelial cells. These nutrient-driven pathways, highlighted in Figure 1, exemplify the translational potential of dietary interventions in cellular aging mitigation.

## 5. Plant-Based Diets and Telomere Biology

Plant-Based diets are dietary patterns that prioritize the consumption of foods derived primarily from plants, including vegetables, fruits, whole grains, legumes, nuts, seeds, and oils, while minimizing or excluding the intake of animal-derived products such as meat, dairy, and eggs [44,45]. These diets emphasize nutrient-dense, fiber-rich foods and are associated with reduced intake of saturated fats and cholesterol. Although the degree of exclusion of animal products can vary—from fully vegan to predominantly plant-centered diets that occasionally include small amounts of animal foods—the core principle remains the emphasis on whole, minimally processed plant foods to support health, prevent chronic diseases, and promote environmental sustainability [44]. Numerous studies have demonstrated that plant-based diets are associated with a lower risk of cardiovascular disease, type 2 diabetes, obesity, and certain types of cancer, as well as potential benefits for longevity and metabolic health [44].

The exploration of dietary influences on aging has increasingly highlighted the role of plant-based diets in modulating telomere dynamics [46]. Multiple observational studies have reported associations between adherence to plant-rich dietary patterns and longer telomere length [46]. Interventional studies have provided more direct evidence of the impact of plant-based diets on telomere biology. Notably, a study led by Dean Ornish demonstrated that a comprehensive lifestyle intervention—including a low-fat, plant-based diet, moderate exercise, stress reduction, and enhanced social support—was associated with increased telomerase activity after three months and longer telomere length at five-year follow-up [47]. Furthermore, the five-year follow-up revealed a significant increase in telomere length among participants adhering to the intervention, compared to telomere shortening in the control group [47]. However, it is also important to emphasize that the observed effects cannot be attributed to the dietary component alone. The multimodal nature of the intervention introduces significant confounding factors, and thus the results should be interpreted as associative rather than causal. Future trials with more isolated dietary manipulations and control of lifestyle variables are required to delineate the independent effects of plant-based diets on telomere biology.

The beneficial effects of plant-based diets on telomere dynamics can be attributed to several nutrient components. Plant-based foods are rich in antioxidants such as vitamins C and E, polyphenols, and carotenoids, which combat oxidative stress—a key contributor to telomere shortening. Compounds like omega-3 fatty acids and flavonoids present in plant foods can reduce chronic inflammation, thereby protecting telomeres from inflammatory damage. Certain plant-derived compounds, including resveratrol and sulforaphane, have been shown to influence epigenetic mechanisms that regulate telomerase activity and telomere maintenance. It is important to note that not all plant-based diets confer the same benefits. A study examining the association of healthy and unhealthy plant-based diets with telomere length found that diets rich in whole, unprocessed plant foods were associated with longer telomeres, whereas those high in refined grains and sugars were linked to shorter telomeres [48].

## 6. Processed and Ultra-Processed Foods: Implications for Telomere Biology and Cellular Aging

The consumption of processed and ultra-processed foods (UPFs) has been increasingly scrutinized for its potential impact on biological aging, particularly concerning telomere dynamics. Processed foods are foods that have been altered from their original form through methods such as canning, freezing, drying, or adding preservatives to enhance shelf life, safety, or palatability. Examples include canned vegetables, cheeses, and freshly made bread. UPFs, a concept defined by the NOVA classification, are industrial formulations made mostly or entirely from substances derived from foods and additives, with little to no intact whole food remaining [49]. They typically contain ingredients such as artificial flavors, colors, emulsifiers, sweeteners, and preservatives. Examples include soft drinks, packaged snacks, instant noodles, and reconstituted meat products. High consumption of UPFs has been associated with increased risks accelerated aging [50].

Several studies have reported associations between high UPF intake and shorter telomere length. A cross-sectional study involving 886 Spanish adults aged 57–91 found that individuals consuming more than three servings of UPFs daily had nearly twice the odds of having shorter telomeres compared to those consuming fewer than two servings per day [51]. Analysis of UK Biobank data from 64,690 participants revealed that each additional daily serving of UPFs was associated with a decrease in leukocyte telomere length (LTL). Participants consuming over eight servings daily exhibited significantly shorter LTL compared to those consuming fewer than 3.5 servings [52]. Not all ultra-processed foods (UPFs) exert uniform effects LTL, as subgroup analyses reveal a nuanced impact depending on the specific type and nutritional profile of the food. Negative associations with LTL have been consistently observed for ready-to-eat or heat-and-serve meals, processed meats, sugary beverages, and products containing artificial sweeteners. Among these, processed meats have emerged as a particularly detrimental category; evidence from the Multi-Ethnic Study of Atherosclerosis indicates that each additional daily serving of processed meat is significantly associated with shorter telomere length [53].

**Figure 2:**
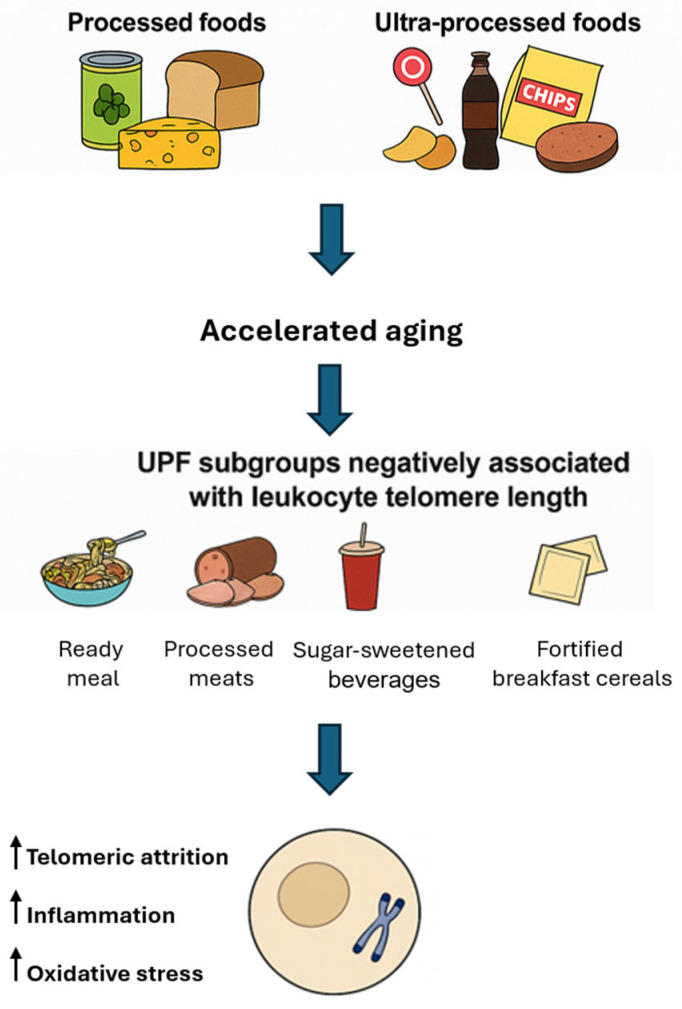
Processed and ultra-processed foods: implications for telomere biology and cellular aging. UPF: ultra-processed food.

The mechanisms underlying the adverse effects of UPFs on telomere integrity likely involve a combination of oxidative stress—driven by high levels of refined sugars and unhealthy fats—inflammation promoted by chemical additives and preservatives, and nutrient deficiencies common in UPF-rich diets, which lack essential vitamins and antioxidants required for telomere maintenance. In contrast to the prevailing association between UPFs and telomere attrition, certain subgroups of UPFs—such as fortified breakfast cereals and some vegetarian alternatives—have shown positive correlations with LTL in observational analyses [54]. This apparent paradox may be explained by the nutritional composition of these items: many breakfast cereals are enriched with B-vitamins and folate, nutrients involved in one-carbon metabolism and DNA methylation [55], which are known to support telomerase activity and telomere maintenance. Similarly, some plant-based meat substitutes are rich in dietary fiber and antioxidants, which may exert anti-inflammatory effects that help preserve telomere integrity. These findings underscore the heterogeneity within the UPF category [56] and highlight the necessity of considering nutritional quality rather than processing level alone when evaluating telomere-related health outcomes. Figure 2 reports on the dietary and mechanistic impacts of processed and UPFs on telomere integrity.

Beyond nutrient deficiencies and pro-oxidative dietary profiles, non-nutritive components of UPFs—such as emulsifiers, colorants, artificial sweeteners, and preservatives—have been shown to exert deleterious effects on gut microbiota composition and function. Experimental studies indicate that additives like carboxymethylcellulose and polysorbate-80 can disrupt the intestinal mucosal barrier, promote dysbiosis, and trigger low-grade systemic inflammation [57]. Artificial sweeteners such as sucralose and aspartame have similarly been associated with altered microbial diversity and pro-inflammatory metabolite production, including lipopolysaccharide (LPS) [58]. These microbiota-mediated pathways contribute to the chronic inflammatory state that accelerates telomere attrition, even in the absence of overt nutritional imbalance [57,58]. Thus, the negative impact of UPFs on telomere biology likely involves a synergistic interaction between nutrient depletion, chemical exposure, and microbiota-derived inflammation.

## 7. Towards a Telomere-Friendly Diet: Key Nutrients and the Power of Plant-Based Nutrition

Considering emerging scientific evidence, a telomere-friendly diet represents a compelling conceptual framework for promoting cellular longevity and mitigating age-related decline. To facilitate individualized planning and guide future research, Table 2 proposes a theoretically tiered intervention model aligned with distinct stages of biological aging. This framework—comprising preventive, therapeutic, and regenerative phases—synthesizes current molecular insights, but does not yet constitute a clinically validated protocol. Its primary objective is to generate hypotheses and inform the design of longitudinal studies and dietary trials focused on telomere biology. In particular, the regenerative phase, targeting older persons with frailty or cognitive impairment, includes interventions such as functional diets and epigenetic supplementation, for which clinical evidence in humans remains preliminary. These components are proposed as research avenues rather than prescriptive treatments and should be interpreted within the context of ongoing translational studies.

The most influential nutrients in this context are those with potent antioxidant, anti-inflammatory, and epigenetic-modulating properties. Plant-based dietary patterns offer an optimal nutrient density for telomere protection. Colorful fruits and vegetables, rich in vitamins C and E, polyphenols, and carotenoids, effectively neutralize oxidative stress—one of the primary accelerators of telomere attrition. Plant-derived omega-3 fatty acids (e.g., from flaxseeds and walnuts), along with flavonoids and soluble fiber, modulate inflammatory responses by inhibiting the NF-κB signaling pathway and reducing pro-inflammatory cytokines such as IL-6 and TNF-α. Beyond vitamin C and resveratrol, the scientific literature reveals several plant-derived bioactive compounds recurrently associated with telomere protection. EGCG (green tea), curcumin (turmeric), quercetin (apples, onions), and sulforaphane (cruciferous vegetables) consistently emerge in both preclinical and observational studies. EGCG modulates epigenetic pathways to upregulate hTERT, curcumin inhibits NF-κB and promotes anti-inflammatory genes, quercetin suppresses IL-6 and TNF-α, and sulforaphane activates Nrf2, thus enhancing antioxidant and detoxification responses. While synergism between nutrients, individual genetic polymorphisms, and lifestyle factors (e.g., physical activity, sleep quality) modulate the bioefficacy of these molecules, their repeated emergence in the literature underscores their centrality in nutritional strategies for telomere maintenance.

Collectively, these components define a nutritional paradigm that not only sustains telomere length but also aligns with broader strategies for healthy aging and disease prevention. Crafting a telomere-friendly diet begins with deliberate food choices that prioritize nutrient density, anti-inflammatory potential, and minimal processing. The cornerstone of this approach lies in whole, plant-based foods that deliver a synergistic array of bioactive compounds. Cruciferous vegetables—such as broccoli, kale, and Brussels sprouts—are rich in sulforaphane and flavonoids, which activate detoxification pathways and protect against DNA damage. Berries, particularly blueberries, strawberries, and blackberries, offer high concentrations of anthocyanins and vitamin C, powerful antioxidants linked to slower telomere attrition. Leafy greens like spinach and arugula provide folate and magnesium, crucial for DNA methylation and cellular repair mechanisms. Nuts and seeds, especially flaxseeds, chia seeds, and walnuts, supply alpha-linolenic acid (ALA), a plant-based omega-3 that supports anti-inflammatory signaling. Whole grains, such as oats, quinoa, and barley, contribute soluble fiber that supports gut microbiota and reduces systemic inflammation. Legumes—lentils, chickpeas, and black beans—are excellent sources of polyphenols and plant-based protein, promoting cellular resilience. Green tea, with its high EGCG content, and turmeric, rich in curcumin, adds functional phytochemicals with proven epigenetic and antioxidant benefits. Minimizing or avoiding ultra-processed foods, sugary snacks, and red or processed meats is essential, as these are consistently associated with shorter telomere length and pro-aging molecular profiles. By integrating these targeted food choices into daily meals, individuals can effectively construct a diet that not only supports telomere maintenance but also enhances overall vitality and healthspan.

## 8. Translational Outlook, Clinical Implications and Future Directions

**Figure 3:**
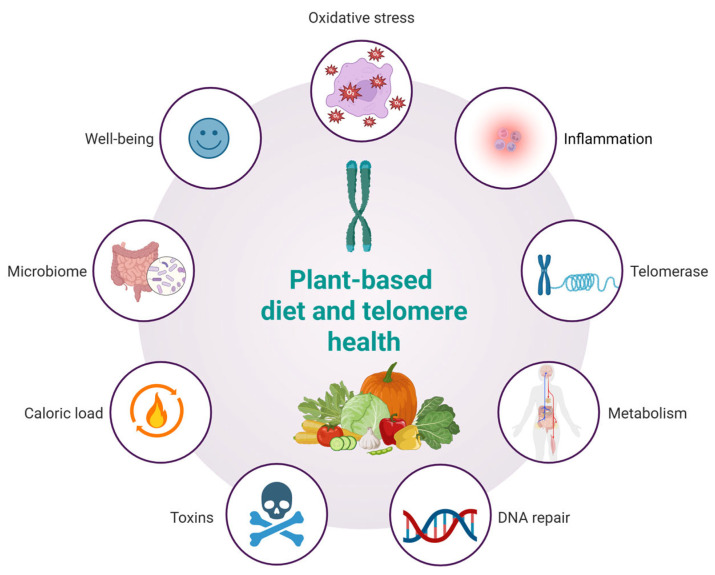
Hypothesized effects of a plant-based diet on telomere health.

A plant-based diet may influence telomere dynamics through multiple interconnected biological pathways (Figure 3). Rich in antioxidants, such diets reduce oxidative stress, thereby limiting DNA damage and preserving telomere length. Anti-inflammatory properties, particularly from fruits, vegetables, and whole grains, may mitigate chronic inflammation known to accelerate telomere attrition. Additionally, improvements in metabolic profiles—such as enhanced insulin sensitivity and lower lipid levels—further protect telomeres. Emerging evidence also suggests potential upregulation of telomerase activity and enhanced DNA repair mechanisms via micronutrient intake. Moreover, plant-based diets reduce exposure to dietary toxins, offer lower caloric density, modulate gut microbiota, and support psychological well-being, all of which may synergistically contribute to telomere maintenance and healthy aging. The concept of a telomere-friendly diet, based on plant-derived nutrients, offers important opportunities for application in geriatric medicine, clinical nutrition, and public health. Since telomere shortening is linked to cellular aging and age-related diseases, dietary strategies that help maintain telomere length are particularly relevant for older adults who are more vulnerable to frailty, chronic inflammation, and oxidative stress. Integrating this dietary model into geriatric care can support more personalized nutrition plans that enhance cellular health. Clinicians and dietitians can use this approach to develop meals rich in polyphenols, omega-3 fatty acids, flavonoids, and nutrients involved in DNA methylation—targeting the key drivers of telomere attrition. This model also aligns well with public health goals to reduce chronic disease risk through diet and lifestyle. Promoting telomere-supportive foods via guidelines, food labeling, and educational campaigns may help improve dietary quality in aging populations. However, while the molecular mechanisms linking plant-based nutrients to telomere preservation have broad biological relevance, their translational validity is not universally applicable in an unqualified manner. The bioefficacy of these compounds is context-dependent, influenced by dietary patterns, metabolic status, genetic polymorphisms (e.g., in hTERT or antioxidant enzymes), gut microbiota composition, and lifestyle factors such as physical activity or sleep quality. For instance, the protective effects of polyphenols may be amplified in Mediterranean-style diets rich in synergistic nutrients but attenuated in diets high in ultra-processed foods that promote oxidative stress and inflammation. Therefore, while the molecular pathways described in this review offer a unifying conceptual framework, their clinical relevance must be interpreted within specific cultural, metabolic, and lifestyle contexts. This nuance highlights the importance of precision nutrition in future interventions targeting healthy aging. While consistent trends link plant-rich diets to longer telomeres, the directionality and causality of these relationships remain unproven due to the influence of confounding variables such as physical activity, stress, sleep, and socioeconomic status. These factors likely act synergistically with diet, and future controlled trials are needed to confirm direct causal effects.

This review adopts a narrative, integrative methodology, which, while suitable for hypothesis generation and conceptual synthesis, is inherently susceptible to selection bias and lacks the systematic rigor of meta-analyses or scoping reviews. The inclusion of studies was guided by relevance to the molecular mechanisms linking plant-based diets and telomere dynamics, but the absence of predefined inclusion/exclusion criteria may limit reproducibility. Moreover, the risk of publication bias must be acknowledged, as studies reporting positive or significant associations between diet and telomere biology are more likely to be published, potentially inflating perceived effect sizes. Additionally, several of the mechanistic insights discussed—particularly those involving epigenetic modulation, oxidative stress, and telomerase activation—derive from preclinical models (in vitro or animal studies) and require further validation in human clinical trials. While the presented tiered intervention model is grounded in molecular plausibility, it remains conceptual and should not be interpreted as a clinically validated framework. These limitations underscore the need for well-designed randomized controlled trials and longitudinal cohort studies to refine our understanding of how specific nutrients, dietary patterns, and lifestyle factors influence telomere biology and aging trajectories. Studies including frailty scores, cognitive function, and inflammatory biomarkers as outcomes would help validate the broader health benefits. However, several questions remain: What are the optimal doses and combinations of bioactive nutrients? Which populations benefit most? How do genetics, metabolism, and gut microbiota influence telomere responses to diet? To address these gaps, future research should include randomized controlled trials, population subgroup analyses, and dose-finding studies. Integrating genomics, epigenetics, and microbiome data could pave the way for precision nutrition strategies tailored to individual biological profiles. Personalized, telomere-focused diets may ultimately improve aging outcomes and enhance the quality of life in older adults. These insights encourage further translational studies integrating nutritional biomarkers, telomere assays, and clinical endpoints in aging populations.

## Figures and Tables

**Table 1:** Main identified putative nutritional modulators of telomere biology.

Nutrient/Compound	Molecular Target/Pathway	Impact on Telomeres
Vitamin C	ROS scavenging, enhances SOD, GPx	Reduces oxidative damage to telomeres
Vitamin E	Lipid membrane stabilization, ROS scavenging	Protects telomeric DNA from peroxidation
Polyphenols	SIRT1 activation, DNA methylation modulation	Enhances telomerase expression, protects telomeres
Omega-3 fatty acids	NF-κB inhibition, IL-6/TNF-α suppression	Reduces inflammation-induced shortening
Curcumin	NF-κB inhibition, antioxidant gene induction	Protects against inflammatory telomere erosion
Flavonoids	Anti-inflammatory cytokine modulation	Reduces SASP and cellular turnover
Folate and B vitamins	One-carbon metabolism, DNA methylation	Supports telomerase gene expression
EGCG (green tea)	Histone acetylation, DNA methylation	Upregulates hTERT, epigenetic stabilization
Sulforaphane	Nrf2 activation, histone deacetylase inhibition	Enhances detoxification, reduces oxidative stress
Nicotinamide riboside	AMPK and SIRT1 activation	Supports mitochondrial health, reduces ROS

ROS, reactive oxygen species; SOD, superoxide dismutase; GPx, glutathione peroxidase; SIRT1, sirtuin 1; NF-κB, nuclear factor kappa-light-chain-enhancer of activated B cells; IL-6, interleukin 6; TNF-α, tumor necrosis factor alpha; SASP, senescence-associated secretory phenotype; hTERT, human telomerase reverse transcriptase; Nrf2, nuclear factor erythroid 2–related factor 2; AMPK, AMP-activated protein kinase.

**Table 2:** Tiered dietary intervention model for telomere integrity.

Phase	Target Group	Objective	Dietary Features
Preventive	Healthy adults (midlife or earlier)	Delay the onset of telomere attrition and systemic inflammaging	- Antioxidant-rich fruits and vegetables - Whole grains, nuts, seeds - Minimized processed sugars and trans fats - Anti-inflammatory spices (e.g., turmeric, ginger) - Folate, vitamin C, omega-3 ALA (e.g., flaxseeds)
Therapeutic	Older adults with mild inflammation, metabolic dysfunction, or early frailty	Halt or reverse inflammation-driven telomere shortening	- Polyphenols (resveratrol, EGCG), omega-3 PUFAs (walnuts, algae oil) - Vitamin D and B-complex supplementation - Soluble fiber for microbiota and CRP modulation - Avoid UPFs and additives - Functional foods (e.g., fermented soy, flaxseed oil)
Regenerative	Older persons with frailty or cognitive decline	Enhance telomerase activity, mitigate SASP, support tissue resilience	- Protein-dense legumes and quinoa - Curcumin + black pepper for synergy - Personalized epigenetic/inflammatory supplementation - High flavonoid intake - Functional and precision diets

ALA, alpha-linolenic acid; EGCG, epigallocatechin gallate; PUFAs, polyunsaturated fatty acids; CRP, C-reactive protein; UPFs, ultra-processed foods; SASP, senescence-associated secretory phenotype.
